# Polygenic risk scores for major psychiatric and neurodevelopmental disorders contribute to sleep disturbance in childhood: Adolescent Brain Cognitive Development (ABCD) Study

**DOI:** 10.1038/s41398-021-01308-8

**Published:** 2021-03-26

**Authors:** Kazutaka Ohi, Ryo Ochi, Yoshihiro Noda, Masataka Wada, Shunsuke Sugiyama, Akira Nishi, Toshiki Shioiri, Masaru Mimura, Shinichiro Nakajima

**Affiliations:** 1 Department of Psychiatry and Psychotherapy, Gifu University Graduate School of Medicine, Gifu, Japan; 2 Department of General Internal Medicine, Kanazawa Medical University, Ishikawa, Japan; 3 Department of Neuropsychiatry, Keio University School of Medicine, Tokyo, Japan

**Keywords:** Clinical genetics, Depression, ADHD, Clinical genetics

## Abstract

Sleep disturbance is a common symptom of psychiatric and neurodevelopmental disorders and, especially in childhood, can be a precursor to various mental disorders. However, the genetic etiology of mental illness that contributes to sleep disturbance during childhood is poorly understood. We investigated whether the polygenic features of psychiatric and neurodevelopmental disorders are associated with sleep disturbance during childhood. We conducted polygenic risk score (PRS) analyses by utilizing large-scale genome-wide association studies (GWASs) (*n* = 46,350–500,199) of five major psychiatric and neurodevelopmental disorders (autism spectrum disorder, schizophrenia, attention-deficit/hyperactivity disorder (ADHD), major depressive disorder (MDD), and bipolar disorder) and, additionally, anxiety disorders as base datasets. We used the data of 9- to 10-year-olds from the Adolescent Brain Cognitive Development study (*n* = 9683) as a target dataset. Sleep disturbance was assessed based on the Sleep Disturbance Scale for Children (SDSC) scores. The effects of PRSs for these psychiatric and neurodevelopmental disorders on the total scores and six subscale scores of the SDSC were investigated. Of the PRSs for the five psychiatric and neurodevelopmental disorders, the PRSs for ADHD and MDD positively correlated with sleep disturbance in children (ADHD: *R*^*2*^ = 0.0033, *p* = 6.19 × 10^−5^, MDD: *R*^*2*^ = 0.0042, *p* = 5.69 × 10^−6^). Regarding the six subscale scores of the SDSC, the PRSs for ADHD positively correlated with both disorders of initiating and maintaining sleep (*R*^*2*^ = 0.0028, *p* = 2.31 × 10^−4^) and excessive somnolence (*R*^*2*^ = 0.0023, *p* = 8.44 × 10^−4^). Furthermore, the PRSs for MDD primarily positively correlated with disorders of initiating and maintaining sleep (*R*^*2*^ = 0.0048, *p* = 1.26 × 10^−6^), followed by excessive somnolence (*R*^*2*^ = 0.0023, *p* = 7.74 × 10^−4^) and sleep hyperhidrosis (*R*^*2*^ = 0.0014, *p* = 9.55 × 10^−3^). Despite high genetic overlap between MDD and anxiety disorders, PRSs for anxiety disorders correlated with different types of sleep disturbances such as disorders of arousal or nightmares (*R*^*2*^ = 0.0013, *p* = 0.011). These findings suggest that greater genetic susceptibility to specific psychiatric and neurodevelopmental disorders, as represented by ADHD, MDD, and anxiety disorders, may contribute to greater sleep problems among children.

## Introduction

Many major psychiatric and neurodevelopmental disorders, such as autism spectrum disorder (ASD), schizophrenia (SCZ), attention-deficit/hyperactivity disorder (ADHD), major depressive disorder (MDD), and bipolar disorder (BIP), can emerge during childhood and adolescence^1^. Indeed, childhood and adolescence are vulnerable periods of brain development, resulting in substantial neurobiological and behavioral changes^2^. Thus, it is reported that the critical stage of neural development is affected by complex interactions among genetic, epigenetic, and environmental factors^3^. However, there are few potential biomarkers for predicting and preventing the onset of these disorders.

Sleep deprivation can cause death more quickly than food deprivation in several species^4^, and the body tries to recover the lost sleep time when prevented from sleeping. Thus, sleep is not simply a period of reduced activity or alertness regulated by circadian rhythms. Sleep is an adaptive behavior that is essential not only for the maintenance of physiological functions, such as metabolic, immune, cardiovascular, and respiratory functions^5,6^, but also for synaptic homeostasis^7^, neuronal recuperation^6^, and brain plasticity^5^, especially in childhood^8^. Sleep supports learning and memory consolidation^9,10^, attention^11^, and emotional processing^12^ and plays a major role in the development of the brain in childhood^8,13^. Sleep architecture undergoes considerable change during development and is vital for brain plasticity, and that there is interindividual variation in sleep^8^. The situational or pathological alterations of sleep can induce maladaptive functioning and psychiatric disorders.

Functional imbalance in the striato-cortical networks is involved in the etiology of insomnia^14,15^. Sleep disturbance is a shared symptom among the previously mentioned psychiatric and neurodevelopmental disorders^16–21^. For example, sleep problems are a core symptom of MDD in children^16^. Sleep disturbances, such as longer latency of sleep onset, nocturnal awakening, and lower sleep efficiency, in children are precursors to MDD^22^. Poor quality of sleep increases the risk of MDD three-fold in children^23^. Additionally, adolescents with ADHD experience more difficulties in initiating and maintaining sleep, and more excessive daytime sleepiness than adolescents without ADHD^17,18^. Additionally, a large-scale epidemiological study in the UK demonstrated the association between the presence of nightmares at age 12 and psychotic experiences at 18^19^. Moreover, several studies have suggested that circadian rhythm dysfunction plays a crucial role in the pathogenesis of BIP^20,24,25^. Furthermore, children with ASD are likely to present with lower melatonin production than children without ASD^21^. Notably, several sleep disturbances precede the onset of core symptoms in psychiatric disorders^1,26,27^. Overall, identifying psychiatric and neurodevelopmental disorder-specific sleep alterations is the first key step to developing preventive action and treatment strategies that can be applied in children’s early life before the onset of these disorders.

The Adolescent Brain Cognitive Development (ABCD) Study is the largest ongoing longitudinal and observational study exploring brain development and child health among children from 21 sites across the United States (https://abcdstudy.org/study-sites/). The ABCD Study has administered baseline comprehensive assessments of the brain development and health of over 10,000 children aged 9–10 years. The relevant data needed for the analysis, including the childhood sleep disturbance scale and genotyping data, are available from the ABCD Study cohort data, which are stored in the National Institute of Mental Health (NIMH) Data Archive (NDA) and are accessible upon request. To date, data from the ABCD Study have indicated that sleep disturbance is positively correlated with depressive symptoms in early adolescents without a diagnosis of MDD^28^.

Psychiatric and neurodevelopmental disorders such as ASD, SCZ, ADHD, MDD, and BIP are common and highly heritable disorders with a polygenic architecture. Several consortia, including the Psychiatric Genomics Consortium (PGC), the Lundbeck Foundation Initiative for Integrative Psychiatric Research (iPSYCH), and the UK Biobank (UKB), have performed large-scale genome-wide association studies (GWASs) of these major psychiatric and neurodevelopmental disorders^29–33^. These GWASs have identified 5, 145, 12, 102, and 30 genetic loci associated with risk for ASD, SCZ, ADHD, MDD, and BIP, respectively. Furthermore, twin studies indicated moderate to strong genetic contributions (*h*^*2*^ = 0.3–0.8) to sleep in adolescence^34^ as well as associations between sleep problems and ADHD in young adulthood^35^, depressive symptoms in young adults^36^, and psychotic experiences in adolescence^37^. Higher heritability of sleep disturbances was indicated in adolescents compared to adults (*h*^*2*^ = 0.3–0.4)^34^. A GWAS has identified more than 200 loci related to insomnia in adults (*n* = 1,331,010), estimated that the variance in the insomnia explained by all GWAS single-nucleotide polymorphisms (SNPs) (SNP-based heritability; *h*^*2*^_SNP_) was 7%, and shown genetic correlations with psychiatric and neurodevelopmental disorders^38^. Sleep disturbance may be a mediated symptom between psychiatric and neurodevelopmental disorders and their susceptibility genes^3^. However, it remains unclear whether genetic risks related to psychiatric and neurodevelopmental disorders are associated with sleep disturbance in prepubertal children. We hypothesized that the large GWAS sample sizes of more than hundreds of thousands of participants would be required to detect genome-wide significant loci related to sleep disturbance in prepubertal children. In contrast, we hypothesized that polygenic risk scores (PRSs) comprising the additive effects of a large number of common SNPs for specific psychiatric and neurodevelopmental disorders would contribute to risk levels of sleep disturbance in prepubertal children; e.g., PRSs for MDD would contribute to disturbance of initiating and maintaining sleep, while PRSs for ADHD would contribute to excessive somnolence.

In the present study, first we preliminarily performed GWASs of the sleep disturbance scale in children (*n* = 9683) from the ABCD Study although the sample sizes were relatively small. Next, we investigated the effects of PRSs for the five major psychiatric and neurodevelopmental disorders (ASD, SCZ, ADHD, MDD, and BIP) on the total scores and six subscale scores ((i) disorders of arousal or nightmares, (ii) disorders of initiating and maintaining sleep, (iii) disorders of excessive somnolence, (iv) sleep breathing disorders, (v) sleep hyperhidrosis, and (vi) sleep–wake transition disorders) of the Sleep Disturbance Scale for Children (SDSC) in children. To replicate the association between PRSs for MDD and sleep disturbance, we further investigated the effects of PRSs for anxiety disorders on the risk of sleep disturbance in children. The PRS analyses were conducted by using data from large-scale GWASs (*n* = 46,350–500,199) of the five major psychiatric and neurodevelopmental disorders and, additionally, anxiety disorders as base datasets and SDSC data for children (*n* = 9683) from the ABCD Study as a target dataset.

## Methods

### Base GWAS datasets

The data from five publicly available GWASs were utilized as base datasets to identify the effective alleles of risk SNPs for each psychiatric and neurodevelopmental disorder, and the *p* values and effect size estimates (odds ratios, ORs) for their associations. Furthermore, the data from GWAS of anxiety disorders were also utilized as base dataset. The GWAS results and sample sizes for five major psychiatric and neurodevelopmental disorders and additional anxiety disorders are shown in Table 1. Here, GWAS summary statistics on five major psychiatric and neurodevelopmental disorders from the PGC, iPSYCH, and UKB (ASD (iPSYCH-PGC GWAS)^29^, SCZ (CLOZUK-PGC2)^30^, ADHD^31^, MDD (PGC-UKB)^32^, and BIP^33^) and anxiety disorders from the UKB^39^ were obtained from a public database (https://www.med.unc.edu/pgc/results-and-downloads; https://ipsych.dk/en/research/downloads/; https://datashare.is.ed.ac.uk/handle/10283/3203; and https://www.kcl.ac.uk/people/kirstin-purves). The detailed sample information regarding the sample collection, genotyping, processing, quality control (QC), and imputation procedures applied in each base GWAS has been described previously^29–33,39^. The sample sizes of the GWASs of these major psychiatric and neurodevelopmental disorders are provided in Table 1.

**Table 1:** Sample sizes for the base and target datasets.

		Sample sizes		
		Total	Cases	Controls^a^
Base GWAS datasets				
ASD	Grove et al. (2019)	46,350	18,381	27,969
SCZ	Pardiñas et al. (2018)	105,318	40,675	64,643
ADHD	Demontis et al. (2019)	55,374	20,183	35,191
MDD	Howard et al. (2019)	500,199	170,756	329,443
BIP	Stahl et al. (2019)	51,710	20,352	31,358
Anxiety disorders	Purves et al. (2020)	83,566	25,453	58,113
Target GWAS datasets				
ABCD (European)	The present study	4920	–	–
ABCD (Trans-ancestry)	The present study	9683	–	–

*ASD* autism spectrum disorder, *SCZ* schizophrenia, *ADHD* attention-deficit/hyperactivity disorder, *MDD* major depressive disorder, *BIP* bipolar disorder, *ABCD* Adolescent Brain Cognitive Development.

^a^Controls or pseudocontrols from trio samples.

### Target GWAS dataset

The ABCD Study is a multisite cohort study with the goal of assessing variability in child and adolescent brain, and cognitive development and understanding factors that influence development from 21 sites across the United States (https://abcdstudy.org/study-sites/). The participants reflect the United States population of boys and girls and include students of diverse races and ethnicities, education and income levels, and living environments. The target dataset consisted of data from 9683 children aged 9–10 years with a mean age of 9.9 years, 52.3% of whom were males (Supplementary Table 1). Of the subjects, 50.5% were participants of European ancestry (*n* = 4920). As over half of the participants in the target GWAS dataset were children of European ancestry (Supplementary Table 1), we first focused on only European participants to avoid population stratification, and then we investigated findings in children of trans-ancestry to generalize the results in European children. These participants were recruited using a school-based recruitment strategy from the ABCD Research Consortium^40^. To ensure the participants met the criteria for current psychiatric and neurodevelopmental disorders, participants and parents were assessed using the Kiddie Schedule for Affective Disorders and Schizophrenia for the DSM-5 (KSADS-5), which is a semistructured interview aimed at the early diagnosis of psychiatric and neurodevelopmental disorders^28,41,42^. The current study used the dataset from the second public release of the baseline ABCD Study data (version 2.0: https://nda.nih.gov/abcd). This study was approved by each local ethical committee of the relevant institutions. All adult participants (parents/caregivers) provided written informed consent, and all youth provided written assent along with permission from a parent/legal guardian.

Saliva was collected from the target subjects, and genomic DNA was extracted. These samples were genotyped on the Affymetrix NIDA SmokeScreen Array (Affymetrix, Santa Clara, CA, USA)^43^. The QC procedures applied in the target dataset are described at the following site: 10.15154/1503209. After QC, 504,943 SNPs were retained. We further checked for sample relatedness using PLINK v1.9, and one of the individuals with close sample relatedness (*pi-hat* > 0.4) was included. Finally, 9683 children, including 4920 individuals of European ancestry, were included in this study (Supplementary Table 1).

The SDSC^44^ was administered to the parents of participants to assess sleep disorders in children over the past 6 months of the child’s life. The SDSC is a 26-item inventory rated on a 5-point Likert-type scale and consists of six sleep disorder subscales. A factor analysis (variance explained 44.2%) yielded six subscales that represented the most common areas of sleep disorders in childhood and adolescence^44^: (i) disorders of arousal or nightmares (DA, three items, sleepwalking, sleep terrors, and nightmares; variance explained 5.9%), (ii) disorders of initiating and maintaining sleep (DIMS, seven items, sleep duration and latency, problems in falling asleep, and night awakenings; variance explained 16.6%), (iii) disorders of excessive somnolence (DOES, five items, daytime somnolence and restless sleep; variance explained 5.1%), (iv) sleep breathing disorders (SBD, three items, sleep apnea and snoring; variance explained 6.3%), (v) sleep hyperhidrosis (SHY, two items, falling asleep sweating and night sweating; variance explained 4.8%), and (vi) sleep–wake transition disorders (SWTD, six items, hypnic jerks, rhythmic movement disorders, and hypnagogic hallucinations; variance explained 5.5%). The total score is the sum of the subscale scores and ranges from 26 to 130 points. A higher score indicates poorer quality of sleep in children.

### Statistical analyses

To exclude the effects of confounding factors on the sleep disturbance scales, we first regressed out the effects of confounding factors, including age, sex, institute, and the first to fourth principal components derived from the genome-wide genotypes to correct for population stratification, on the sleep disturbance scales, and yielded confounding factor-corrected and unstandardized scores of the SDSC using R 3.5.0 (http://www.r-project.org/). For the preliminary GWASs of the sleep disturbance scales, we excluded SNPs that (1) had a low genotype call frequency of <0.95, (2) were localized on the Y chromosome or mitochondria, (3) deviated from Hardy-Weinberg equilibrium (*p* < 1.0 × 10^−5^), or (4) had a low minor allele frequency (MAF) of <0.01 among children of European ancestry and children of trans-ancestry, respectively. The preliminary GWASs were conducted under a linear regression model with the corrected total scores of the SDSC as a dependent variable and the additive genotype dosages as independent variables using PLINK v1.9. SNP heritability of the corrected scales of sleep disturbance was calculated using the package GCTA (Genome-wide Complex Trait Analysis; GCTA v1.93; https://cnsgenomics.com/software/gcta/)^45^. To remove SNPs that were in linkage disequilibrium (LD) in the base and target GWAS datasets, the SNPs were pruned based on a pairwise *r*^2^ threshold of 0.10 and a window size of 250 kb using PRSice-2^46^. We then calculated PRSs constructed from SNPs showing a nominal association with each psychiatric and neurodevelopmental disorder and anxiety disorders in the base GWASs under the following five liberal significance thresholds (*P*_T cutoff_): *P*_T_ ≤ 0.01, *P*_T_ ≤ 0.05, *P*_T_ ≤ 0.1, *P*_T_ ≤ 0.5, and *P*_T_ ≤ 1 using PRSice-2. The number of SNPs used for PRS analyses at each *P*_T cutoff_ is provided in Supplementary Table 2. For each participant included in the target dataset, a PRS was calculated by weighting the scores for ‘risk SNPs’ by the logarithm of the OR (logOR) observed in each base GWAS dataset. The score, consisting of the number of risk alleles (0, 1, or 2) multiplied by the logOR, was summed over all of the SNPs in the *P*_T_-SNP sets for each individual in the target dataset. To examine the effects of the PRSs for the major psychiatric and neurodevelopmental disorders and anxiety disorders on the risk level of sleep disturbance in children given each *P*_T cutoff_, we performed linear regression with the corrected total scores or subscale scores of the SDSC as dependent variables, and each PRS based on each GWAS of the psychiatric and neurodevelopmental disorders and anxiety disorders as independent variables. We compared the differences in the adjusted *R*^2^, which is a measure of the variance explained by the model. As the PRSs at each *P*_T cutoff_ were highly correlated with each other and were not independent, the *p* values based on different *P*_T cutoff_ values were not corrected. A conservative Bonferroni-corrected *p* value threshold of *p* < 0.01 (=0.05/5 psychiatric and neurodevelopmental disorders) was used to avoid type I error.

## Results

### GWAS of sleep disturbance in children

The distributions of raw total scores on the sleep disturbance scale in children of European ancestry (*n* = 4920) and children of trans-ancestry (*n* = 9683) are presented in Supplementary Fig. 1. These distributions were similar between children of European ancestry and children of trans-ancestry. Phenotypic correlations among the total scores and six subscale scores of the SDSC and ADHD symptoms assessed by the Child Behavior Checklist (CBCL) are shown in Supplementary Table 3. Each subscale was weakly to modestly correlated with each other (all *p* < 0.001). Symptoms of ADHD were weakly to modestly correlated with sleep disturbance scales (all *p* < 0.001), particularly disorders of initiating and maintaining sleep, and disorders of excessive somnolence. We preliminarily performed GWASs of the total scores of the sleep disturbance scale in children of European ancestry and children of trans-ancestry (Supplementary Fig. 2). As expected, we did not find any genome-wide significant SNPs associated with sleep disturbance (Supplementary Fig. 2, *p* > 5.0 × 10^−8^), except for two SNPs (rs41383545, beta ± SE = 3.5 ± 0.5, *p* = 3.09 × 10^−12^ and rs115710069, beta ± SE = 3.2 ± 0.5, *p* = 8.59 × 10^−10^) in trans-ancestry populations; however, the MAF of these two SNPs was <0.01 in children of European ancestry. The top marginally associated SNPs (*p* < 1.00 × 10^−5^) in these GWASs are summarized in Supplementary Table 4. In contrast, the PRSs obtained from the GWAS of sleep disturbance in children of European ancestry were positively correlated with sleep disturbance in children of other ethnicities (maximum at *P*_T_ ≤ 0.1, *R*^*2*^ = 0.0016, *p* = 6.23 × 10^−3^), suggesting a genetic architecture of sleep disturbance shared between European and non-European children. SNP heritability (*h*^*2*^_SNP_) of the total scores and six subscale scores of the SDSC is shown in Supplementary Fig. 3. The SNP heritability based on all GWAS SNPs was significantly estimated from 5.5% for SWTD to 22.9% for DOES in children of trans-ancestry (*p* < 0.05). In contrast, these GWAS SNPs could not significantly explain variances in SBD or SHY in children of European ancestry and children of trans-ancestry (*p* > 0.05).

### Effects of PRSs for psychiatric and neurodevelopmental disorders on the risk of sleep disturbance in children

**Fig. 1:**
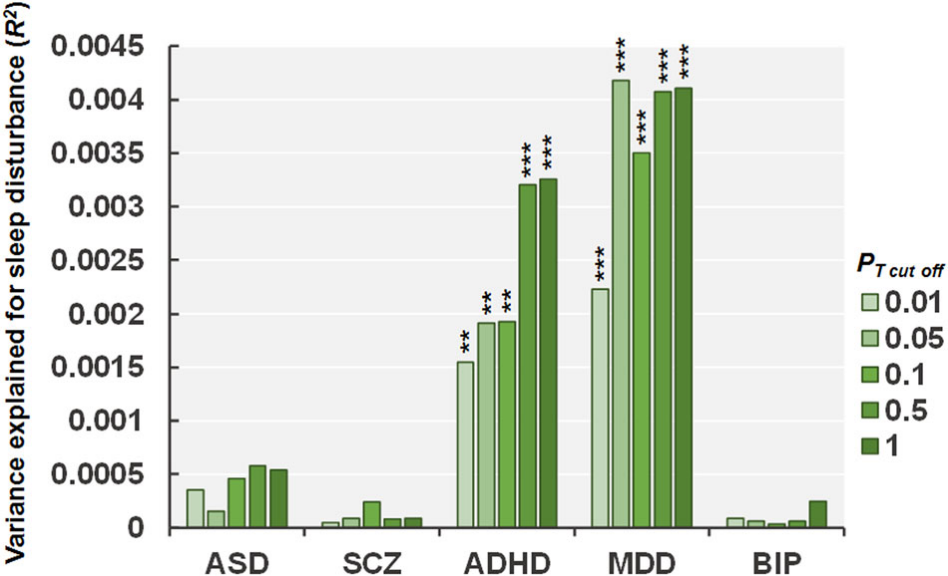
Effects of polygenic risk scores (PRSs) for psychiatric and neurodevelopmental disorders. Effects of polygenic risk scores (PRSs) for psychiatric and neurodevelopmental disorders (ASD, SCZ, ADHD, MDD, and BIP) at each threshold (*P*_T cutoff_) on the risk of sleep disturbance in early adolescence in children of European ancestry. The *y*-axis represents the adjusted *R*^2^, indicating the explanatory power of the model. ASD, autism spectrum disorder; SCZ, schizophrenia; ADHD, attention-deficit/hyperactivity disorder; MDD, major depressive disorder; BIP, bipolar disorder. ***p < 0.001, **p < 0.01, *p < 0.05.

**Fig. 2:**
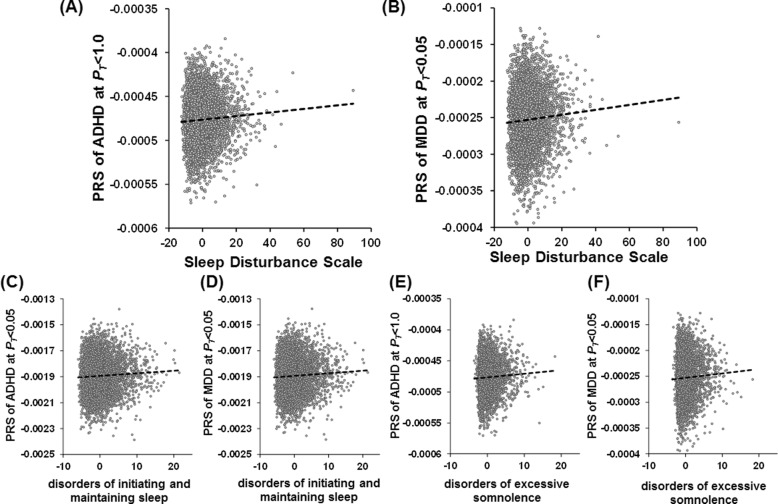
Positive correlations between PRSs based on GWASs of ADHD and MDD. Positive correlations between PRSs based on GWASs of ADHD and MDD, and the total scale (**A**, **B**) and subscales (**C**–**F**) of childhood sleep disturbance in European children. Confounding factor-corrected total scores and subscale scores of the sleep disturbance scale are indicated. The PRSs for each psychiatric and neurodevelopmental disorder at *P*_T cutoff_ with the most significant correlations with sleep disturbance are indicated.

To examine whether children with higher genetic risks related to psychiatric and neurodevelopmental disorders would have higher or lower sleep disturbance in childhood, we investigated the effects of PRSs for the five psychiatric and neurodevelopmental disorders (ASD, SCZ, ADHD, MDD, and BIP) on the risk levels of the total scores of the SDSC in European children (*n* = 4920) at different *P*_T cutoff_ levels (Fig. 1). The PRSs for ADHD and MDD were significantly correlated with sleep disturbance in European children (Figs. 1 and 2; ADHD: maximum at *P*_T_ ≤ 1.0, *R*^*2*^ = 0.0033, *p* = 6.19 × 10^−5^; MDD: maximum at *P*_T_ ≤ 0.05, *R*^*2*^ = 0.0042, *p* = 5.69 × 10^−6^). The PRSs related to both ADHD and MDD were positively correlated with sleep disturbance (Fig. 2). We reviewed these findings after including diagnostic status (ADHD (*n* = 448) or MDD (*n* = 8)) as a covariate or excluding these patients from the sample. Ever after diagnostic status was considered, polygenic risks for both ADHD and MDD were still significantly associated with sleep disturbance (*p* < 0.05), indicating that the PRS results were not affected by the inclusion of these patients in the sample. Even after children of other ethnicities were included, these findings remained significant among children of trans-ancestry (*n* = 9683) (Supplementary Fig. 4). In contrast, there were no significant correlations between the PRSs for ASD, SCZ, or BIP and the SDSC total scores in European children or children of trans-ancestry (*p* > 0.05).

### Effects of PRSs for psychiatric and neurodevelopmental disorders on the scores of the six subscales of sleep disturbance in children

**Fig. 3:**
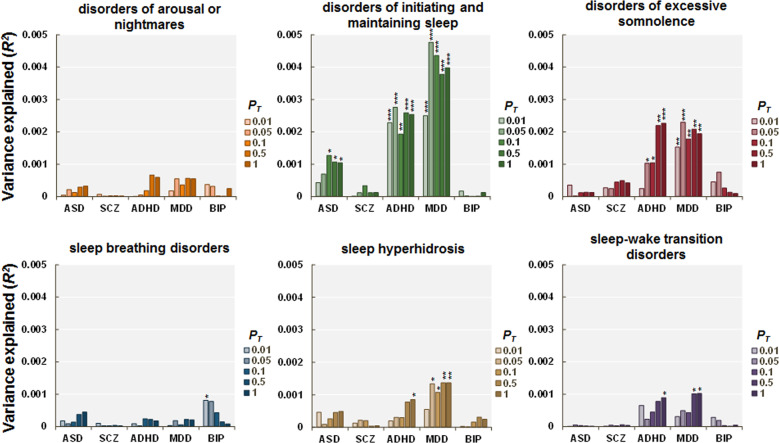
Effects of PRSs for psychiatric and neurodevelopmental disorders. Effects of PRSs for psychiatric and neurodevelopmental disorders on the risk of six types of sleep disturbance in early adolescence in European children. ***p < 0.001, **p < 0.01, *p < 0.05.

We next investigated the effects of PRSs for psychiatric and neurodevelopmental disorders on the scores of the six subscales of the SDSC ((i) disorders of arousal or nightmares, (ii) disorders of initiating and maintaining sleep, (iii) disorders of excessive somnolence, (iv) sleep breathing disorders, (v) sleep hyperhidrosis, and (vi) sleep–wake transition disorders) in European children (Figs. 2 and 3). Of the six subscales, the PRSs for ADHD were positively correlated with disorders of initiating and maintaining sleep (maximum at *P*_T_ ≤ 0.05: *R*^*2*^ = 0.0028, *p* = 2.31 × 10^−4^) and disorders of excessive somnolence (maximum at *P*_T_ ≤ 1.0: *R*^*2*^ = 0.0023, *p* = 8.44 × 10^−4^) in European children. Similarly, the PRSs for MDD were positively correlated with disorders of initiating and maintaining sleep (maximum at *P*_T_ ≤ 0.05: *R*^*2*^ = 0.0048, *p* = 1.26 × 10^−6^), disorders of excessive somnolence (maximum at *P*_T_ ≤ 0.05: *R*^*2*^ = 0.0023, *p* = 7.74 × 10^−4^), and sleep hyperhidrosis (maximum at *P*_T_ ≤ 0.5: *R*^*2*^ = 0.0014, *p* = 9.55 × 10^−3^) in European children.

Furthermore, the PRSs for ADHD were weakly, positively correlated with sleep hyperhidrosis (maximum at *P*_T_ ≤ 1.0: *R*^*2*^ = 0.0009, *p* = 0.041) and sleep–wake transition disorders (maximum at *P*_T_ ≤ 1.0: *R*^*2*^ = 0.0009, *p* = 0.037). The PRSs for ASD were weakly, positively correlated with disorders of initiating and maintaining sleep (maximum at *P*_T_ ≤ 0.1: *R*^*2*^ = 0.0013, *p* = 0.012). The PRSs for BIP were weakly, positively correlated with sleep breathing disorders (maximum at *P*_T_ ≤ 0.01: *R*^*2*^ = 0.00081, *p* = 0.046). However, these weak correlations were not significant after Bonferroni correction was applied (*p* > 0.01). There were no significant correlations between the PRSs obtained from GWASs of other psychiatric and neurodevelopmental disorders and the other subscales of the SDSC (*p* > 0.05). Even after children of other ethnicities were included, these findings did not change in children of trans-ancestry (*n* = 9683) (Supplementary Fig. 5).

### Effects of PRSs for anxiety disorders on the risk of sleep disturbance in children

**Fig. 4:**
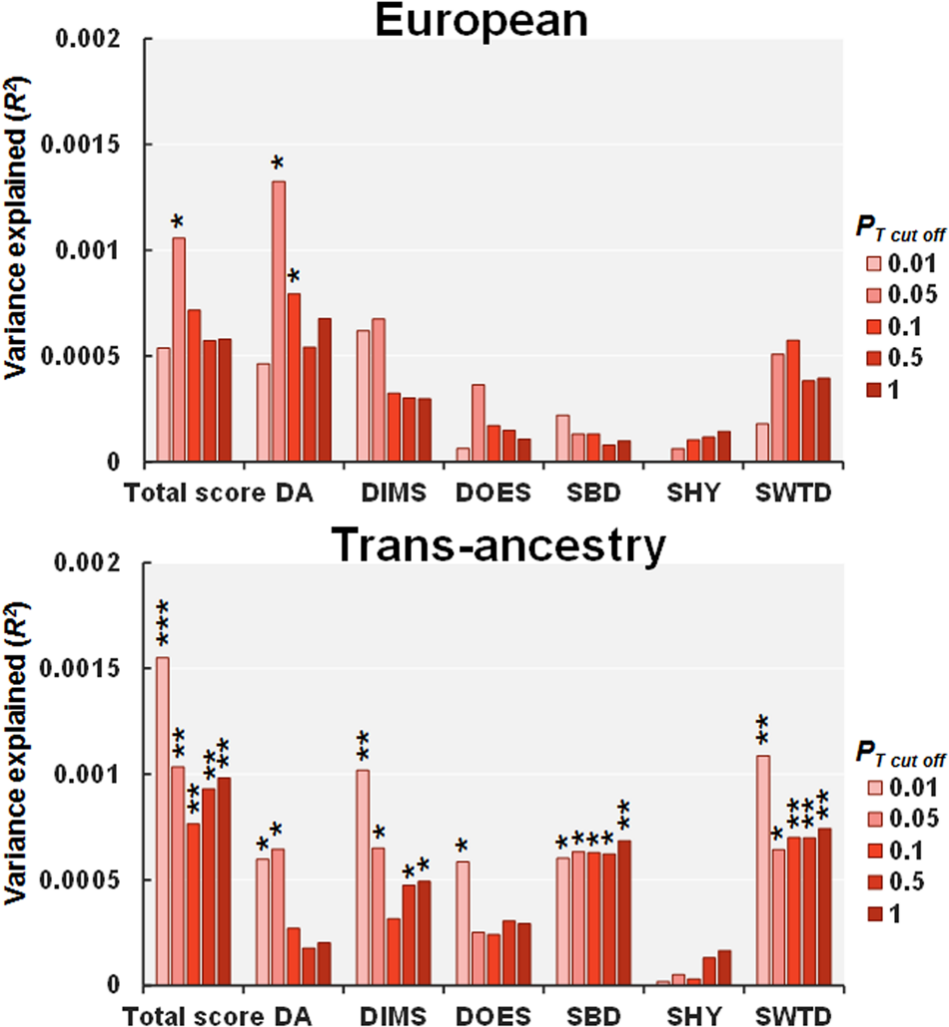
Effects of PRSs for anxiety disorders on the risk of sleep disturbance in young adolescence in European children and in children of trans-ancestry. Effects of PRSs for anxiety disorders on the risk of sleep disturbance in young adolescence in European children and in children of trans-ancestry. ***p < 0.001, **p < 0.01, *p < 0.05. DA, disorders of arousal or nightmares; DIMS, disorders of initiating and maintaining sleep; DOES, disorders of excessive somnolence; SBD, sleep breathing disorders; SHY, sleep hyperhidrosis; SWTD, sleep–wake transition disorders.

There is a high genetic overlap between MDD and anxiety disorders (*r*_g_ > 0.80)^39,47^. Sleep disturbance is also a core symptom of anxiety disorders, and sleep disturbance appears before the anxiety disorders in some patients^48^. Although examining anxiety disorders was not part of our original analysis plan, we added the phenotype as a result of a peer-review process. Therefore, based on the high shared-genetic risk and phenotypic overlap between MDD and anxiety disorders, we hypothesized that PRSs for anxiety would be correlated with sleep disturbance particularly in disorders of initiating and maintaining sleep. We attempted to replicate the relationship between PRSs for MDD and sleep disturbance using proxy PRSs based on GWAS of anxiety disorders (Fig. 4). The PRSs for anxiety disorders were positively correlated with sleep disturbance in both European children (maximum at *P*_T_ ≤ 0.05, *R*^*2*^ = 0.0011, *p* = 0.023) and children of trans-ancestry (maximum at *P*_T_ ≤ 0.01, *R*^*2*^ = 0.0016, *p* = 1.06 × 10^−4^). Of the six subscales, the PRSs for anxiety disorders were positively correlated with disorders of arousal or nightmares in both European children (maximum at *P*_T_ ≤ 0.05, *R*^*2*^ = 0.0013, *p* = 0.011) and children of trans-ancestry (maximum at *P*_T_ ≤ 0.05, *R*^*2*^ = 0.0006, *p* = 0.012). Furthermore, the PRSs for anxiety disorders were positively correlated with disorders of initiating and maintaining sleep (maximum at *P*_T_ ≤ 0.01, *R*^*2*^ = 0.0010, *p* = 1.69 × 10^−3^), disorders of excessive somnolence (maximum at *P*_T_ ≤ 0.01, *R*^*2*^ = 0.0006, *p* = 0.017), sleep breathing disorders (maximum at *P*_T_ ≤ 1.0, *R*^*2*^ = 0.0007, *p* = 9.99 × 10^−3^), and sleep–wake transition disorders (maximum at *P*_T_ ≤ 0.01, *R*^*2*^ = 0.0011, *p* = 1.18 × 10^−3^) only in children of trans-ancestry.

## Discussion

This is the first study to investigate whether the polygenic risk components for major psychiatric and neurodevelopmental disorders (ASD, SCZ, ADHD, MDD, and BIP) and anxiety disorders contribute to the sleep disturbance of children aged 9–10 years using PRS analyses. Of the five major psychiatric and neurodevelopmental disorders, we revealed that there were significant genetic overlaps of sleep disturbance in prepubertal children with the polygenic risk for ADHD and MDD. Higher polygenic risks for both ADHD and MDD were associated with greater sleep disturbance in children. Furthermore, higher polygenic risk for ADHD was equally associated with disorders of initiating and maintaining sleep and disorders of excessive somnolence in children, while higher polygenic risk for MDD was mainly associated with disorders of initiating and maintaining sleep, followed by disorders of excessive somnolence and sleep hyperhidrosis. Despite high genetic correlation between MDD and anxiety disorders, higher polygenic risk for anxiety disorders was associated with different types of sleep disturbances such as disorders of arousal or nightmares. These findings suggest that genetic factors related to ADHD, MDD, and anxiety disorders could contribute to specific sleep disorders in children.

Adolescents with ADHD and MDD typically report sleep complaints, such as daytime sleepiness and sleep deprivation^49,50^. These sleep problems are not secondary to the disorders; rather, sleep complaints generally precede the onset of these psychiatric and neurodevelopmental disorders in children and adolescents^1^. As expected, although the number of patients was limited, European children with ADHD (*n* = 448) and MDD (*n* = 8) displayed greater sleep disturbance than those without ADHD and MDD (*p* < 0.05). Even after the diagnostic status was considered, PRSs for both ADHD and MDD were associated with sleep disturbance. Our main findings suggest that the PRSs for ADHD and MDD may explain a portion of the etiology of sleep disturbance regardless of current diagnostic status. Furthermore, reduced cortical areas, which are more strongly determined by genetic influences^51^, are specifically associated with insomnia severity, not with depression severity in MDD^52^. Given these findings, sleep disturbance may play a role in the intermediation between genetic risk and the onset of these psychiatric and neurodevelopmental disorders, e.g., via reduced cortical areas. Therefore, identifying the genetic risks of ADHD and MDD in combination with sleep disturbance in children could facilitate the development of predictive and preventative tools for the onset of these psychiatric and neurodevelopmental disorders.

To detect disorder-specific sleep problem patterns preceding the onset of ADHD and MDD, examining individual items of the SDSC subscales that are related to polygenic risks for these disorders is of particular importance. Children with a higher genetic risk for ADHD exhibited more symptoms of both disorders of initiating and maintaining sleep (insomnia) and disorders of excessive somnolence (hypersomnia). Similarly, a higher genetic risk for MDD was mainly associated with insomnia, followed by hypersomnia and sleep hyperhidrosis in childhood. These symptoms are consistent with clinical features of sleep disturbance in these disorders. These three subscales of the SDSC may be useful to detect children at risk of developing ADHD and MDD. Furthermore, improvements in the quality of sleep during childhood may contribute to ongoing neurodevelopmental processes facilitating and maintaining normal neuroplasticity, which may decrease the impact of neural vulnerabilities to ADHD and MDD, and reduce the risk of onset for these disorders.

Common genetic effects on anxiety disorders are highly shared with MDD^39,47^, and sleep disturbance is a common symptom of both disorders^48^. Consistent with the relationship between PRSs for MDD and sleep disturbance, we confirmed that higher PRSs for anxiety disorders were associated with greater sleep disturbance in children. However, the PRSs for anxiety disorders were correlated with disorders of arousal or nightmares of the six subscales, while the PRSs for MDD were not correlated with the arousal disorders. Sleep problems, such as nightmares, sleep terrors, and nocturnal panic attacks, are related to anxiety disorders^48^. Nightmares mostly occur during REM sleep, while nocturnal panic attacks generally occur during late stage 2 to early stage 3 sleep, and sleep terrors mostly occur during stage 4 sleep^48^. Nocturnal panic attack which refers to waking from sleep in a panic state should be distinguished from nighttime arousal induced by nightmares and it has often been mistaken for sleep apnea and sleep terrors. The systems involving corticotropin-releasing hormone and locus ceruleus–autonomic nerves play major roles in the arousal response to stress, and these systems may be vulnerable to prolonged or repeated stress^48^. Alterations in these systems may lead to a dysfunctional arousal state and pathological anxiety states in anxiety disorders. Therefore, arousal disorders related to PRSs for anxiety disorders might be derived from nightmares, sleep terrors, or nocturnal panic attacks. We suggest that the genetic risks of MDD and anxiety disorders might contribute to different sleep problems regardless of high genetic overlap between disorders.

Using the same sample, Goldstone et al. demonstrated that sleep disturbance, such as disorders of excessive somnolence, predicted depressive symptoms at the 1-year follow-up as well as baseline depressive symptoms^28^. Subjects participating in the current study were identical to these 9- and 10-year-old subjects, but only one of the individuals with close sample relatedness, such as that between twins and siblings, among these subjects was included in the current study. Based on a clinical interview with youth and parents^28^, a few subjects who met the criteria for current MDD (*n* = 18) were included in the present study. Therefore, our findings, including the current diagnosis of MDD, were not influenced by sampling bias. To further establish when and how sleep disturbance facilitates the onset of these psychiatric and neurodevelopmental disorders, longitudinal analyses using upcoming ABCD Study releases should be conducted in future research.

We could not detect significant associations of the sleep disturbance with the PRSs for ASD, SCZ, or BIP. Compared with effects of PRSs for ADHD, MDD, and anxiety disorders on the risk of sleep disturbance, effects of PRSs for ASD, SCZ, and BIP were small. The main weakness of the current study is the status of the existing base GWAS datasets. Sample sizes in the base datasets varied among psychiatric and neurodevelopmental disorders, ranging from 46,350 for ASD to 500,199 for MDD (Table 1). As the statistical power in the PRS analysis depends on the sample sizes in the base GWAS datasets^53,54^, we could not completely exclude possible effects of smaller base sample sizes on our null findings, such as for ASD. Therefore, comparing the impacts of the PRSs on the sleep disturbance across disorders should be carefully interpreted for heterogeneous data since its sample sizes were not uniform across disorders.

As the genetic risks for psychiatric and neurodevelopmental disorders are thought to be shared with each other, we explored correlations across PRSs for psychiatric and neurodevelopmental disorders at *P*_T_ ≤ 1.0 in young adolescence in European children (Supplementary Fig. 6). The PRSs were weakly correlated with each other (all *p* < 0.001), e.g., *r* = 0.19 between ADHD and MDD. Despite the difference in the sample sizes in the base GWAS, we additionally examined a multi-PRS model with all the PRSs for ASD, SCZ, ADHD, MDD, BIP, and anxiety disorders at *P*_T_ ≤ 1.0 in order to find which effects are more specific for sleep disturbance. Higher polygenic risks for ADHD and MDD were independently associated with greater sleep disturbance in children of European ancestry and children of trans-ancestry (*p* < 0.01), while other PRSs for ASD, SCZ, BIP, and anxiety disorders were not strongly associated with sleep disturbance (*p* > 0.01). Furthermore, higher polygenic risk for ADHD was mainly associated with disorders of excessive somnolence (*p* < 0.01), while higher polygenic risk for MDD was mainly associated with disorders of initiating and maintaining sleep (*p* < 0.01).

We found that genetic risks for psychiatric and neurodevelopmental disorders were associated with sleep disturbance in prepubertal children. As some subjects who met the criteria for current ADHD were included, we further tested whether (i) PRSs for ADHD were associated with risk of ADHD in children of European ancestry and children of trans-ancestry and (ii) the association was mediated by sleep disturbance using a regression analysis with ADHD-PRS as the independent variable, a sleep disturbance measure as the mediator, and ADHD diagnosis as the dependent variable. We confirmed that PRSs for ADHD were significantly associated with risk of ADHD and the association was attenuated when the sleep disturbance was included in the analysis. These findings suggest that sleep disturbance in prepubertal children could be one of the mediators between at least ADHD and their susceptibility genes.

There are some limitations that should be considered in the interpretation of our findings. Since the severity of sleep disturbance in children was assessed based on subjective parental perceptions, further research using objective measurements of sleep disturbance is needed. According to the original and subsequent studies^44^, we used the identical terms of six subscales of the SDSC. However, the SDSC does not definitely measure sleep ‘disorders’ but provide trait/symptom dimensions. The diagnostic information used in this study was also collected from parents, necessitating further studies. Our main findings suggest that the PRSs for ADHD, MDD, and anxiety disorders explain a portion of the variance; approximately 0.33%, 0.42%, and 0.11%, in the sleep disturbance, respectively. These findings are surely statistically significant, but the small effect sizes mean that their clinical significance would be quite limited. We did not examine gene–environment interactions. Our PRS associations might be affected by some environmental factors, such as parental socioeconomic status^55^ and parental mental status. Thus, there is potential indirect effect of genetic risks for psychiatric and neurodevelopmental disorders on sleep disturbance in children. Although we found that genetic risks were associated with sleep disturbance, it remains unclear whether the sleep disturbance would be cause or consequence of disorders. To test for putative causal association between sleep disturbance and these disorders, further study with larger GWAS datasets using a multi-SNP Mendelian randomization analysis^56^ is required.

## Conclusion

We investigated shared genetic backgrounds between sleep disturbance in children and children’s risks of major psychiatric and neurodevelopmental disorders and anxiety disorders. Based on the mounting evidence that individuals with ADHD, MDD, and anxiety disorders are often accompanied by sleep disturbance^16–18,48^, our findings further confirmed that genetic vulnerabilities to ADHD, MDD, and anxiety disorders positively correlate with sleep disturbance in childhood. In particular, genetic vulnerability to ADHD is associated with both insomnia and hypersomnia, while genetic vulnerability to MDD is associated with mainly insomnia, followed by hypersomnia and sleep hyperhidrosis. Furthermore, genetic vulnerability to anxiety disorders is associated with arousal disorder. Taken together with clinical evidence, identifying the disorder-specific sleep alterations in children would be useful for the early detection and prevention of ADHD, MDD, and anxiety disorders. Further longitudinal research of sleep disturbance from childhood to young adulthood might contribute to the understanding of the genetic architecture underlying ADHD, MDD, and anxiety disorders as well as to early detection and prevention for children at risk for these disorders.

## Supplementary information

Supplementary information.
