# Supplementary material for: Polygenic risk scores for major psychiatric and neurodevelopmental disorders contribute to sleep disturbance in childhood: Adolescent Brain Cognitive Development (ABCD) Study
Source: Transl Psychiatry. 2021 Mar 26;11:187. doi: 10.1038/s41398-021-01308-8 (PMC7997961; doi:10.1038/s41398-021-01308-8)
Supplement: Supplementary file 1 — Supplementary information. [file 41398_2021_1308_MOESM1_ESM.docx]

**Supplementary Information**

**Supplementary Table 1.** Demographic information for the target GWAS dataset

|  | **European** | **Trans-ancestry** |
| --- | --- | --- |
|  | **(*n*=4,920)** | **(*n*=9,683)** |
| **Age, month (mean ± SD)** | 119.5±7.5 | 119.1±7.5 |
| **Sex (male/female)** | 2,639/2,281 | 5,068/4,615 |
| **Ethnicity (*n*)** |  |  |
| Caucasian | 4,920 | 4,920 |
| African | - | 1,455 |
| Hispanic | - | 2,034 |
| Asian | - | 210 |
| Others | - | 1,064 |
| **Current diagnosis (+/-/NA)** |  |  |
| ASD or other neurodevelopmental disorder | 1,310/3,552/58 | 2,629/6,910/144 |
| SCZ or other psychotic disorder | 9/4,861/50 | 26/9,534/123 |
| ADHD | 448/4,416/56 | 856/8,689/138 |
| MDD | 8/4,860/52 | 18/9,536/129 |
| BIP | 7/4,861/52 | 27/9,527/129 |

**Supplementary Table 2.** Number of SNPs used for the PRS analyses at each *P_T_* *_cutoff_* in the target dataset

|  |  | **Base** | **Number of SNPs used for analyses at *P_T_* in target sample** | | | | |
| --- | --- | --- | --- | --- | --- | --- | --- |
|  |  | **SNPs (*n*)** | **0.01** | **0.05** | **0.1** | **0.5** | **1** |
| ASD | Grove *et al.* (2019) | 6,517,324 | 3,227 | 11,695 | 20,670 | 71,324 | 109,424 |
| SCZ | Pardiñas *et al*. (2018) | 7,578,889 | 7,851 | 19,004 | 28,624 | 76,067 | 109,956 |
| ADHD | Demontis *et al.* (2019) | 8,047,420 | 3,464 | 11,516 | 19,451 | 62,450 | 92,765 |
| MDD | Howard *et al.* (2019) | 8,483,301 | 5,756 | 16,114 | 25,758 | 76,170 | 113,637 |
| BIP | Stahl *et al.* (2019) | 9,629,760 | 4,622 | 14,959 | 25,312 | 83,319 | 127,248 |
| Anxiety | Purves *et al.* (2019) | 7,926,782 | 3,146 | 12,099 | 21,526 | 75,421 | 116,360 |

ASD, autism spectrum disorder; SCZ, schizophrenia; ADHD, attention-deficit/hyperactivity disorder; MDD, major depressive disorder; BIP, bipolar disorder.

**Supplementary Table 3.** Phenotypic correlations among the total scores and six subscale scores of the Sleep Disturbance Scale for Children (SDSC) and ADHD symptoms in children of European ancestry and children of trans-ancestry.

|  | **Total** | **DA** | **DIMS** | **DOES** | **SBD** | **SHY** | **SWTD** | **CBCL** |
| --- | --- | --- | --- | --- | --- | --- | --- | --- |
| Total scores | - | 0.45 | 0.80 | 0.71 | 0.39 | 0.43 | 0.71 | 0.43 |
| DA | 0.51 | - | 0.24 | 0.19 | 0.14 | 0.20 | 0.34 | 0.17 |
| DIMS | 0.79 | 0.28 | - | 0.47 | 0.12 | 0.20 | 0.32 | 0.36 |
| DOES | 0.73 | 0.27 | 0.47 | - | 0.19 | 0.17 | 0.33 | 0.32 |
| SBD | 0.45 | 0.19 | 0.16 | 0.24 | - | 0.18 | 0.30 | 0.14 |
| SHY | 0.47 | 0.23 | 0.21 | 0.21 | 0.22 | - | 0.29 | 0.16 |
| SWTD | 0.73 | 0.41 | 0.34 | 0.37 | 0.31 | 0.33 | - | 0.30 |
| CBCL | 0.42 | 0.20 | 0.36 | 0.33 | 0.15 | 0.17 | 0.30 | - |

Pearson's correlation coefficients in children of European ancestry (above) and children of trans-ancestry (below) are represented. DA, disorders of arousal or nightmares; DIMS, disorders of initiating and maintaining sleep; DOES, disorders of excessive somnolence; SBD, sleep breathing disorders; SHY, sleep hyperhidrosis; SWTD, sleep-wake transition disorders; CBCL, Child Behavior Checklist. ADHD symptoms were assessed by using the DSM-Oriented Scales (DOS) of the CBCL that is a standardized questionnaire. The parents were asked to report about the children’s behavior using the CBCL.

**Supplementary Table 4.** Top SNPs (*p*<1.0×10^-5^) in the GWASs of the sleep disturbance scale in European children and in children of trans-ancestry

|  |  |  |  |  | **European** | | | | **Trans-ancestry** | | | | **Nearest** |
| --- | --- | --- | --- | --- | --- | --- | --- | --- | --- | --- | --- | --- | --- |
| **SNP** | **Chr** | **Bp** | **A1** | **A2** | **MAF(A1)** | ***n*** | ***β±SE*** | ***p*** | **MAF(A1)** | ***n*** | ***β±SE*** | ***p*** | **gene** |
| **GWAS in European children** | | | | | | | | | | | | | |
| rs8019773 | 14 | 44891480 | C | T | 0.33 | 4,698 | -0.8±0.2 | 1.47×10^-6^ | 0.30 | 9,253 | -0.3±0.1 | 0.011 | *LINC02277* |
| rs41292768 | 13 | 51508967 | G | T | 0.028 | 4,678 | 2.3±0.5 | 1.69×10^-6^ | 0.027 | 9,221 | 1.0±0.4 | 8.73×10^-3^ | *RNASEH2B* |
| rs1366636 | 5 | 36585689 | G | A | 0.20 | 4,697 | 0.9±0.2 | 2.38×10^-6^ | 0.18 | 9,259 | 0.6±0.2 | 5.20×10^-5^ | *SLC1A3* |
| rs4151664 | 6 | 31920873 | T | C | 0.055 | 4,699 | 1.6±0.3 | 2.58×10^-6^ | 0.074 | 9,261 | 1.1±0.2 | 2.81×10^-6^ | *NELFE* |
| rs28676679 | 8 | 131511982 | A | G | 0.40 | 4,692 | 0.7±0.2 | 3.62×10^-6^ | 0.37 | 9,248 | 0.3±0.1 | 0.010 | *ASAP1* |
| rs12362554 | 11 | 79134808 | A | G | 0.19 | 4,701 | 0.9±0.2 | 4.52×10^-6^ | 0.23 | 9,258 | 0.5±0.1 | 5.77×10^-4^ | *TENM4* |
| rs2731905 | 5 | 36567156 | T | C | 0.20 | 4,672 | 0.9±0.2 | 4.71×10^-6^ | 0.19 | 9,198 | 0.6±0.2 | 9.50×10^-5^ | *SLC1A3* |
| rs73188628 | 3 | 187721148 | G | T | 0.14 | 4,693 | 1.0±0.2 | 4.98×10^-6^ | 0.12 | 9,242 | 0.6±0.2 | 3.11×10^-3^ | *LOC107986166* |
| rs12478477 | 2 | 52311667 | A | G | 0.28 | 4,700 | 0.8±0.2 | 5.44×10^-6^ | 0.24 | 9,262 | 0.4±0.1 | 2.11×10^-3^ | *LOC730100* |
| rs62102601 | 19 | 31820573 | T | C | 0.075 | 4,701 | -1.3±0.3 | 6.31×10^-6^ | 0.052 | 9,268 | -0.9±0.3 | 1.31×10^-3^ | *TSHZ3* |
| rs508446 | 1 | 111309830 | C | T | 0.22 | 4,690 | -0.8±0.2 | 8.66×10^-6^ | 0.22 | 9,245 | -0.5±0.1 | 1.06×10^-3^ | *LOC105378901* |
| **GWAS in children of trans-ancestry** | | | | | | | | | | | | | |
| rs41383545 | 4 | 142677910 | A | G | <0.01 | - | - | - | 0.013 | 9,258 | 3.5±0.5 | **3.09×10^-12^** | *IL15* |
| rs115710069 | 7 | 112966027 | A | G | <0.01 | - | - | - | 0.012 | 9,230 | 3.2±0.5 | **8.59×10^-10^** | *SMIM30* |
| rs11977139 | 7 | 22347641 | G | A | <0.01 | - | - | - | 0.013 | 9,169 | 2.5±0.5 | 1.23×10^-6^ | *RAPGEF5* |
| rs4151664 | 6 | 31920873 | T | C | 0.055 | 4,699 | 1.6±0.3 | 2.58×10^-6^ | 0.074 | 9,261 | 1.1±0.2 | 2.81×10^-6^ | *NELFE* |
| rs117161424 | 20 | 61343602 | G | T | 0.072 | 4,697 | 0.9±0.3 | 2.27×10^-3^ | 0.056 | 9,255 | 1.2±0.3 | 3.92×10^-6^ | *NTSR1* |
| rs73811895 | 3 | 10406969 | T | C | <0.01 | - | - | - | 0.012 | 9,267 | 2.4±0.5 | 5.40×10^-6^ | *ATP2B2* |
| rs76641358 | 4 | 103078138 | T | C | <0.01 | - | - | - | 0.012 | 9,261 | -2.3±0.5 | 5.51×10^-6^ | *BANK1* |
| rs6097888 | 20 | 52899581 | T | C | <0.01 | - | - | - | 0.013 | 9,144 | 2.3±0.5 | 6.68×10^-6^ | *PFDN4* |
| rs2675653 | 10 | 63606563 | A | G | 0.047 | 4,703 | 0.8±0.4 | 0.023 | 0.055 | 9,263 | 1.2±0.3 | 7.81×10^-6^ | *LINC02625* |
| rs4789376 | 17 | 74929998 | A | C | 0.36 | 4,689 | 0.4±0.2 | 7.37×10^-3^ | 0.37 | 9,226 | 0.5±0.1 | 9.96×10^-6^ | *MGAT5B* |

Chr, chromosome; Bp, nucleotide location; A, allele; MAF, minor allele frequency; SE, standard error. Marginally associated SNPs (*p*<1.0×10^-5^) in the GWASs of the sleep disturbance scale in European children and in children of trans-ancestry are summarized. Genome-wide significant *p* values are shown as bold font and are underlined.

**
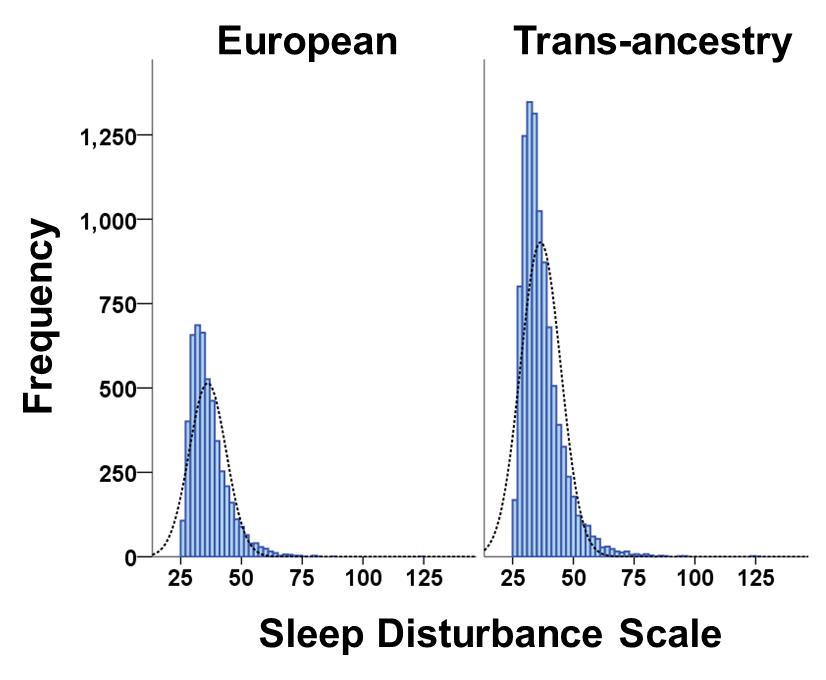
**

**Supplementary Figure 1.** The distributions of the raw total scores of the sleep disturbance scale in European children (*n*=4,920) and children of trans-ancestry (*n*=9,683).

**
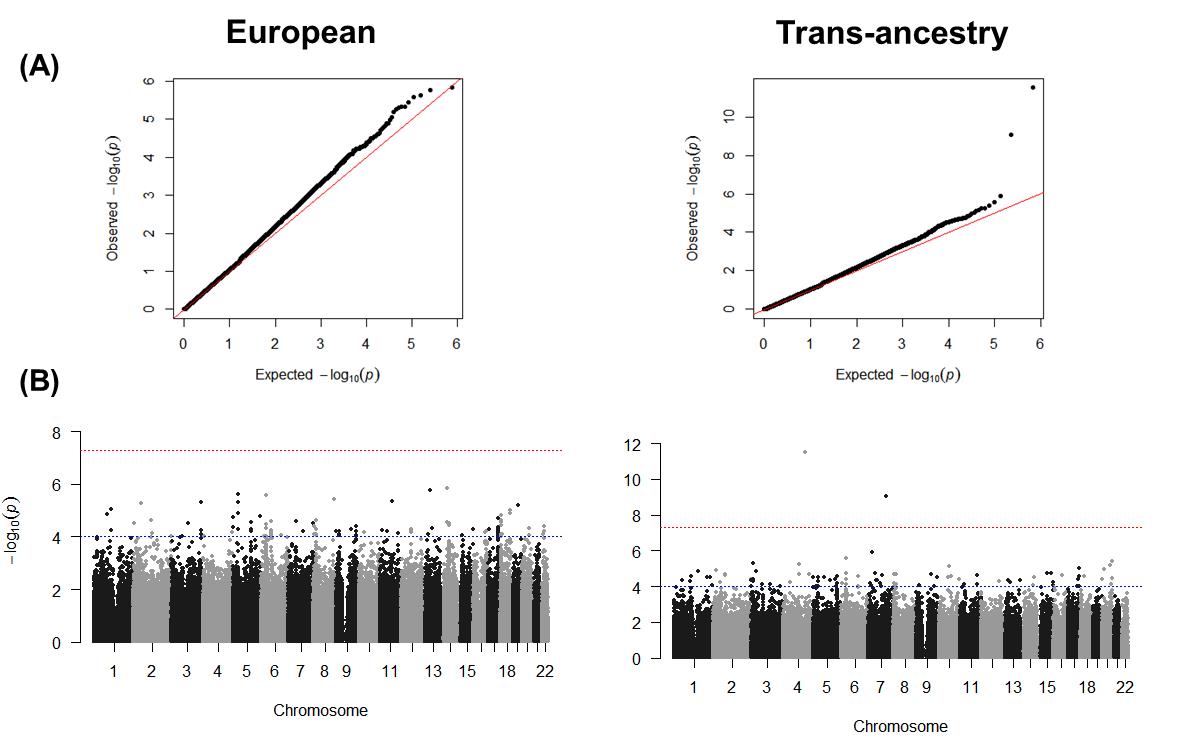
**

**Supplementary Figure 2.** GWASs of the sleep disturbance scale in European children and in children of trans-ancestry. QQ plots (A) and Manhattan plots (B) derived from the GWASs of sleep disturbance. The blue dotted lines in the Manhattan plots indicate a *p* value of 1.0×10^-4^. The red dotted lines indicate a *p* value of 5.0×10^-8^.

**
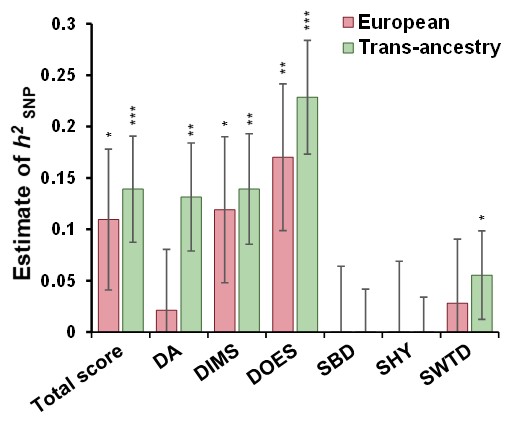
**

**Supplementary Figure 3.** Estimates of variance explained by all autosomal SNPs for total scores and the scores of six subscales of the SDSC (SNP heritability; *h^2^*_SNP_). Error bars represent the SE of the estimates. ^***^*p*<0.001, ^**^*p*<0.01, ^*^*p*<0.05. DA, disorders of arousal or nightmares; DIMS, disorders of initiating and maintaining sleep; DOES, disorders of excessive somnolence; SBD, sleep breathing disorders; SHY, sleep hyperhidrosis; SWTD, sleep-wake transition disorders.


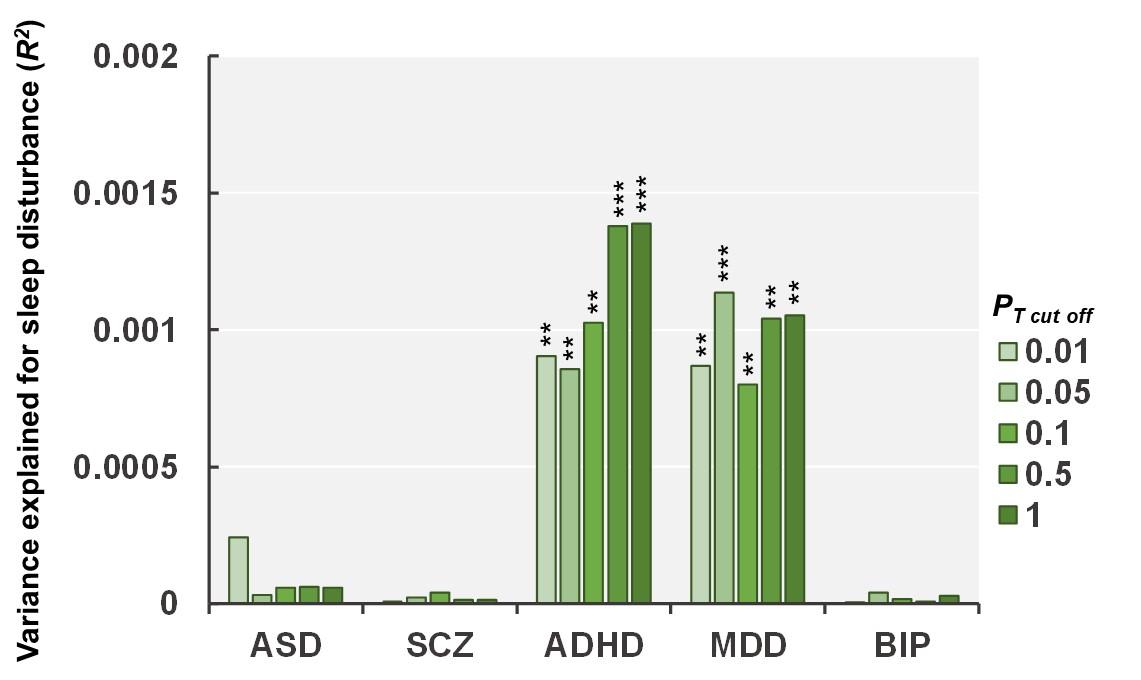


**Supplementary Figure 4.** Effects of PRSs for psychiatric and neurodevelopmental disorders on the risk of sleep disturbance in young adolescence in children of trans-ancestry. ^***^*p*<0.001, ^**^*p*<0.01, ^*^*p*<0.05.


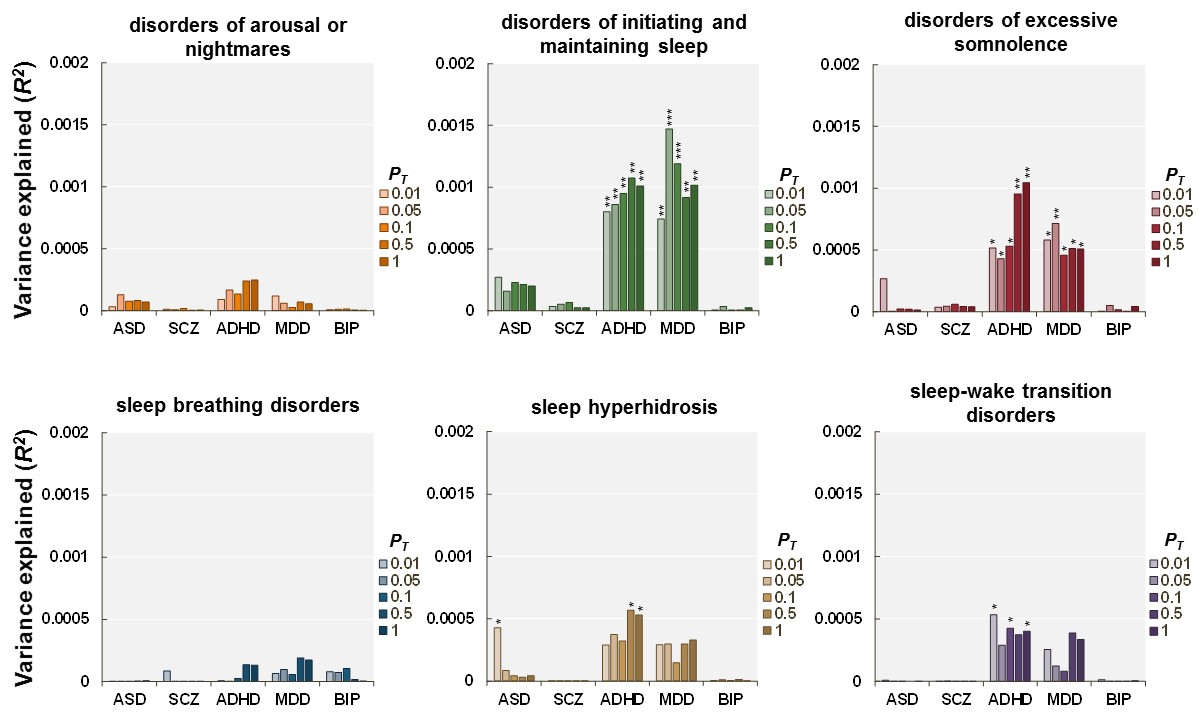


**Supplementary Figure 5.** Effects of PRSs for psychiatric and neurodevelopmental disorders on the risk of six types of sleep disturbance in young adolescence in children of trans-ancestry. ^***^*p*<0.001, ^**^*p*<0.01, ^*^*p*<0.05.


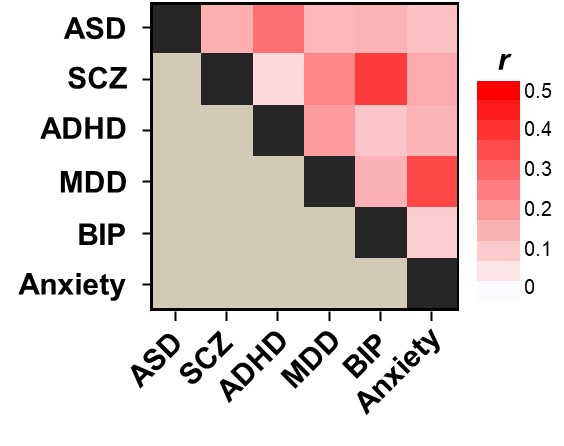


**Supplementary Figure 6.** Correlations across PRSs for psychiatric and neurodevelopmental disorders at *P_T_*≤1.0 in young adolescence in European children (all *p*<0.001). The color scale represents the Pearson's *r*. ASD, autism spectrum disorder; SCZ, schizophrenia; ADHD, attention-deficit/hyperactivity disorder; MDD, major depressive disorder; BIP, bipolar disorder.
